# Acral Melanoma in an Irish Population: Clinicopathological Features, Molecular Profile and Implications for Early Detection

**DOI:** 10.3390/dermatopathology13040046

**Published:** 2026-10-01

**Authors:** Mary Ellen McMahon, Stephanie M. Bollard, John M. Keaney, Linda Kelly, Jean Paul Coetser, Silvia Marino, Michael O’Connor, Chloe Spillane, Donal Murphy, Aurelie Fabre, Barry O’Sullivan, Arnold D. K. Hill, Fiachra Martin, Roisin T. Dolan

**Affiliations:** 1 Department of Plastic and Reconstructive Surgery, Beaumont Hospital, Dublin 9, Ireland; 2 Department of Plastic and Reconstructive Surgery, St. Vincent’s University Hospital, Dublin 4, Ireland; 3 School of Medicine, Royal College of Surgeons in Ireland, Dublin 2, Ireland; 4 Department of Pathology, St. Vincent’s University Hospital, Dublin 4, Ireland; 5 UCD School of Medicine & Medical Sciences, University College Dublin, Dublin 4, Ireland; 6 Department of Surgery, Beaumont Hospital, Dublin 9, Ireland

**Keywords:** acral melanoma, acral lentiginous melanoma, delayed diagnosis, nail unit melanoma, BRAF, triple-wild-type melanoma, digit-sparing surgery, sentinel lymph node biopsy

## Abstract

Acral melanoma (AM) is a rare, biologically distinct melanoma subtype arising on the palms, soles, and nail apparatus that is frequently diagnosed late and carries a worse prognosis than non-acral cutaneous melanoma. Population-specific AM data remain limited outside North America and East Asia, and Ireland’s comparatively homogeneous population has not previously been described. We performed a retrospective, bi-institutional cohort study of 41 AM patients treated at two Dublin tertiary referral centres (2017–2023), using data from prospectively maintained melanoma databases, medical records, and pathology reports. Patients were predominantly older adults (median age 73, range 39–88) with a long delay before presentation: 80.5% reported an enlarging lesion for six months or more before seeking care. Lesions were predominantly located on the foot (73.2%), and acral lentiginous melanoma was the predominant histological subtype (82.9%). Median Breslow Thickness was 3.0 mm, and 75.6% presented with T stage ≥ 3. Amputation was required in 65.9% of patients to achieve adequate margins, 47.4% of those assessed had confirmed nodal disease, and 36.6% developed recurrence. Mutation testing, performed in a subset of tumours, identified BRAF, NRAS, and KIT alterations in a minority of cases, with a substantial proportion classified as triple-wild-type. Despite Ireland’s unique germline background reported in cutaneous melanoma, this Irish cohort’s mutational profile and advanced stage at presentation were broadly consistent with international cohorts. Earlier clinical recognition, standardised nail-unit biopsy technique, and a national melanoma registry could improve outcomes for this rare, high-risk subtype.

## Simple Summary

Acral melanoma is a rare skin cancer that develops on the palms, soles, and around the nails, areas not usually exposed to the sun. Because it often does not look like a typical mole and can be mistaken for a wart, a fungal infection, or a minor injury, it is frequently diagnosed late, when treatment options are more limited. We reviewed the records of 41 patients treated for acral melanoma at two hospitals in Dublin, Ireland, over six years. Most patients had noticed a changing mark for six months or more before seeing a doctor, and most were diagnosed with thick, advanced tumours that required amputation of part of a finger or toe. We describe the typical features of this cancer in an Irish population for the first time and outline practical steps that can help doctors and patients recognise it earlier.

## 1. Introduction

Melanoma accounts for 1.8% of new cancer diagnoses worldwide annually [1], and its incidence is increasing across Europe by an average of 3–4% per year [2]. Acral melanoma (AM) is a rare but clinically aggressive form of melanoma. It is defined by its location, arising on non-hair-bearing acral surfaces, including the palms, volar surfaces of digits, plantar aspect of the feet and toes, and periungual/nail matrix [3,4]. Unlike acral lentiginous melanoma, which is a specific histological subtype, AM encompasses all melanomas in these acral locations and may include NM, SSM, amelanotic, desmoplastic and ALM [5].

Unlike other cutaneous melanomas, AM shows a markedly weaker association with Ultraviolet (UV) exposure. BRAF, NRAS and KIT mutations, which are common in UV-driven melanoma, occur less frequently, while mechanisms related to chronic trauma and mechanical stress have been proposed to contribute to its pathogenesis [6].

AM also presents a well-documented diagnostic challenge. Acral sites are common locations for benign or traumatic mimics, such as diabetic ulceration, tinea pedis, pressure ulcers, or abrasions. The traditional triage algorithms for pigmented lesions, such as the ABCDE rule, perform poorly in this setting, with misdiagnosis rates of approximately 30% reported [7]. Consequently, late diagnoses at advanced disease stage and more aggressive surgical management are commonplace [8]. As a result, the 5-year melanoma-specific survival rate of 80% to 81% for ALM, compared to 91% to 93% for non-ALM cutaneous melanoma [9], making early recognition and prompt management of AM crucial to minimise morbidity and optimise patient survival.

Much of the current understanding of AM derives from cohorts in North America, continental Europe, and Asia, though mutational profiles are known to vary across geographic region and ancestry [10]. Ireland offers a distinctive population in which to examine this. It is comparatively genetically homogeneous, with a high prevalence of melanocortin-1 receptor (MC1R) variants associated with fair skin and red hair [11,12]. This variant has been associated with an increased risk of developing invasive melanoma in the context of oncogenic BRAF activation [13]. A lower rate of BRAF mutations has also been observed in the Irish population compared with international reported data [14]. Whether this distinctive germline background is reflected in the mutational profile of acral melanoma has not been previously examined. Describing AM within an Irish cohort is therefore important to determine whether international observations are applicable in this population and establishes a baseline against which future national and collaborative data can be compared.

This retrospective, bi-institutional cohort study describes the demographic, clinicopathological, molecular, and treatment-related characteristics of patients with AM treated at two tertiary melanoma referral centres in Ireland. The aims were to characterise the clinical and molecular profile of AM in an Irish population, compare these features with other published cohorts, and highlight practice-relevant implications for earlier recognition, biopsy, and surgical management of AM.

## 2. Materials and Methods

### 2.1. Study Design and Setting

This study is reported in accordance with the Strengthening the Reporting of Observational Studies in Epidemiology (STROBE) guidelines for cohort studies (Supplementary Materials). A retrospective cohort study was conducted of patients diagnosed with and/or treated for AM between January 2017 and December 2023 at two tertiary referral centres for melanoma in Dublin, Ireland. Together, these institutions serve a catchment population of approximately 2 million, close to 40% of the national population [15]. Ethical approval was obtained from the Beaumont Hospital Ethics (Medical Research) Committee (Ref: 24/52, October 2024) and was registered as a clinical audit with the Clinical Audit Committee of St. Vincent’s University Hospital (Ref: 3924, December 2023).

Patients were eligible for inclusion if they had a diagnosis of AM confirmed on histopathology, defined as melanoma arising on non-hair-bearing acral skin of the palms, soles, volar surfaces of the digits, or nail apparatus, and were managed at either participating institution during the study period. No additional exclusion criteria were applied. As cutaneous melanoma is managed only in specialist tertiary referral centres in our jurisdiction, case capture is expected to be comprehensive, with selection bias considered to be limited.

### 2.2. Data Collection and Study Variables

Data were extracted from prospectively maintained melanoma databases, medical records, radiology reports, and histopathology reports. Patient-level variables included age, sex, ethnicity, tumour location, clinical pigmentation status, duration of symptoms prior to presentation, and referral source. Histopathological features included Breslow Thickness (BT), Tumour stage according to AJCC 8th edition (T stage), histological subtype, the presence of ulceration, perineural invasion (PNI), and Lymphovascular Invasion (LVI). Treatment variables comprised surgical management, sentinel lymph node biopsy (SLNBx) and completion lymph node dissection where performed, adjuvant systemic therapy, disease recurrence, and survival status at last follow-up. Due to the variation in follow-up, formal survival estimation was not performed, and overall survival is reported as the crude percentage alive at last follow-up. Where mutation testing (BRAF, NRAS, KIT) had been performed as part of routine clinical care, results were recorded, and tumours negative for all three were classified as triple-wild-type (TWT). Patients were followed up according to standard stage-appropriate protocols, comprising clinical review and radiological surveillance where indicated, with outcomes censored in December 2024.

Data were analysed using IBM SPSS Statistics (v 29.0) [16]. Categorical variables are reported as absolute frequency and percentage of the available denominator, and continuous variables are reported as mean with standard deviation (SD) or median with range. Percentages were calculated using the number of patients with available data as the denominator, and missing values were not imputed and are indicated where relevant. No formal sample size calculation was performed. All patients meeting the inclusion criteria in the study period were included.

## 3. Results

### 3.1. Patient Characteristics

Forty-one patients met the inclusion criteria. There was near-equal sex distribution (male, *n* = 19, 46.3%; female, *n* = 22, 53.7%). Median age at diagnosis was 73 years (range 39–88 years). All patients were recorded as being of Caucasian ethnicity. The majority of patients (*n* = 33, 80.5%) observed presence of a lesion for ≥ 6 months before self-referring to a primary care physician.

Lesions were located on the foot in 30/41 patients (73.2%), while the remaining 11 were located on the hand (26.8%). The most common single site was the plantar foot (*n* = 14, 34.1%), followed by the hallux (*n* = 9, 22.0%). Full anatomical distribution and clinicopathological characteristics are summarised in Table 1.

### 3.2. Clinicopathological and Molecular Characteristics

**Figure 1:**
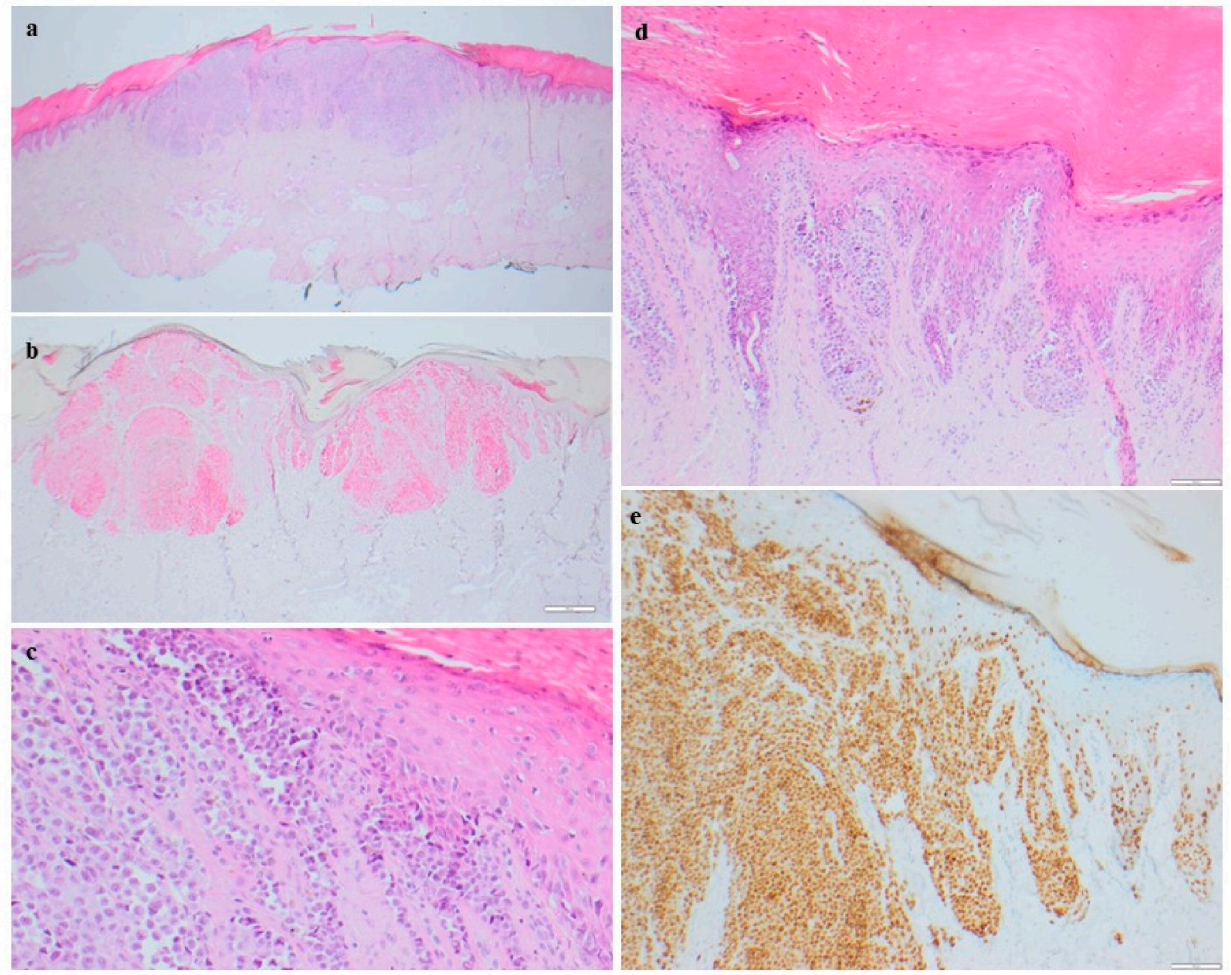
Histological appearance of an ulcerated acral melanoma. (**a**) Low-power view of acral melanoma with central ulceration (×1.25 magnification, haematoxylin and eosin stain). (**b**) Corresponding MelA immunostaining (×2 magnification). (**c**,**d**) Closer appearance with invasion into the dermis (**c**) and pagetoid spread of malignant melanocytes into the epidermis (**d**) (×20 and ×10 magnification, haematoxylin and eosin stain). (**e**) PRAME immunohistochemistry positive in both epidermal and dermal components (×10 magnification).

Acral lentiginous melanoma was the predominant histological subtype of AM, identified in 34/41 (82.9%; Figure 1), followed by nodular melanoma in *n* = 5 (12.2%). Superficial spreading and spindle cell melanomas each accounted for a single case. Median BT was 3 mm (range 0.65–38.00 mm), and 75.6% of patients (31/41) presented with T stage ≥ 3.

Clinical pigmentation data were complete for all 41 patients. Clinically amelanotic or hypopigmented lesions were reported in *n* = 17 (41.5%), and *n* = 24 (58.5%) demonstrated clinical pigmentation. Ulceration was present in *n* = 17 patients, with ulceration status available for 39/41 patients, giving a rate of 43.6% among those with data available. Lymphovascular Invasion was present in *n* = 8 patients, and PNI in *n* = 6. The median mitotic rate was 3.0 mitoses per mm^2^ (range 0–40), regression was present in *n* = 2, and microsatellites were present in *n* = 5.

Mutation testing had been performed in a subset of *n* = 23 tumours. Of those, BRAF mutations were most frequent (*n* = 7), followed by NRAS (*n* = 5) and KIT (*n* = 4). Where specified, BRAF mutations comprised V600E (*n* = 2), V600G (*n* = 1), and the non-V600 variant D594G (*n* = 1), and KIT mutations included D816Y (*n* = 1) and L576P (*n* = 1). One tumour tested positive for mutations in BRAF, NRAS, and KIT, and nine tumours were classified as TWT and negative for all three.

### 3.3. Treatment and Outcomes

Amputation was required in 27/41 patients (65.9%) to achieve oncologically adequate margins. In total, 38/41 patients (92.7%) underwent some form of nodal assessment, of whom *n* = 18 (47.4% of those assessed) had histologically confirmed nodal disease. SLNBx was performed in 35/41 patients (85.4%), of whom *n* = 15 (42.9% of those biopsied, 36.6% of cohort) had a positive result. A further three patients (7.3%) presented with clinically palpable nodal disease confirmed by core biopsy. Completion lymphadenectomy was performed in 12 patients (eight inguinal, four axillary).

Disease recurrence occurred in 36.6% of patients (*n* = 15), with interval from diagnosis to recurrence ranging from 8 to 85 months. The first site of recurrence was local in *n* = 7, regional nodal disease in *n* = 3, in-transit in *n* = 1, and distant metastatic disease in *n* = 4, comprising the lung, liver, and brain.

Systemic therapy was administered to 29.3% of patients (*n* = 12), either in the adjuvant setting following wide local excision (*n* = 9) or following recurrence (*n* = 9). A total of *n* = 6 patients received systemic therapy in both settings. The predominant approach was single-agent anti-PD-1 therapy (nivolumab, *n* = 8; pembrolizumab, *n* = 2), with escalation to combination ipilimumab and nivolumab in *n* = 3 patients following disease progression. One patient whose tumour harboured a BRAF mutation received dabrafenib and trametinib, and individual patients received tumour-infiltrating lymphocyte therapy and temozolomide. The specific agent was not recorded in one patient.

Follow-up data were available for *n* = 35 patients, with a median follow-up of 45 months (range 12–110). At last follow-up, 23 of these 35 patients were alive, resulting in a crude overall survival of 65.7%. Among the *n* = 12 deaths in the follow-up period, the cause of death was melanoma-specific in *n* = 6 patients, non-melanoma-related in *n* = 2 (including a second-primary lung adenocarcinoma), and undetermined in *n* = 4.

## 4. Discussion

### 4.1. Disease Characteristics

**Figure 2:**
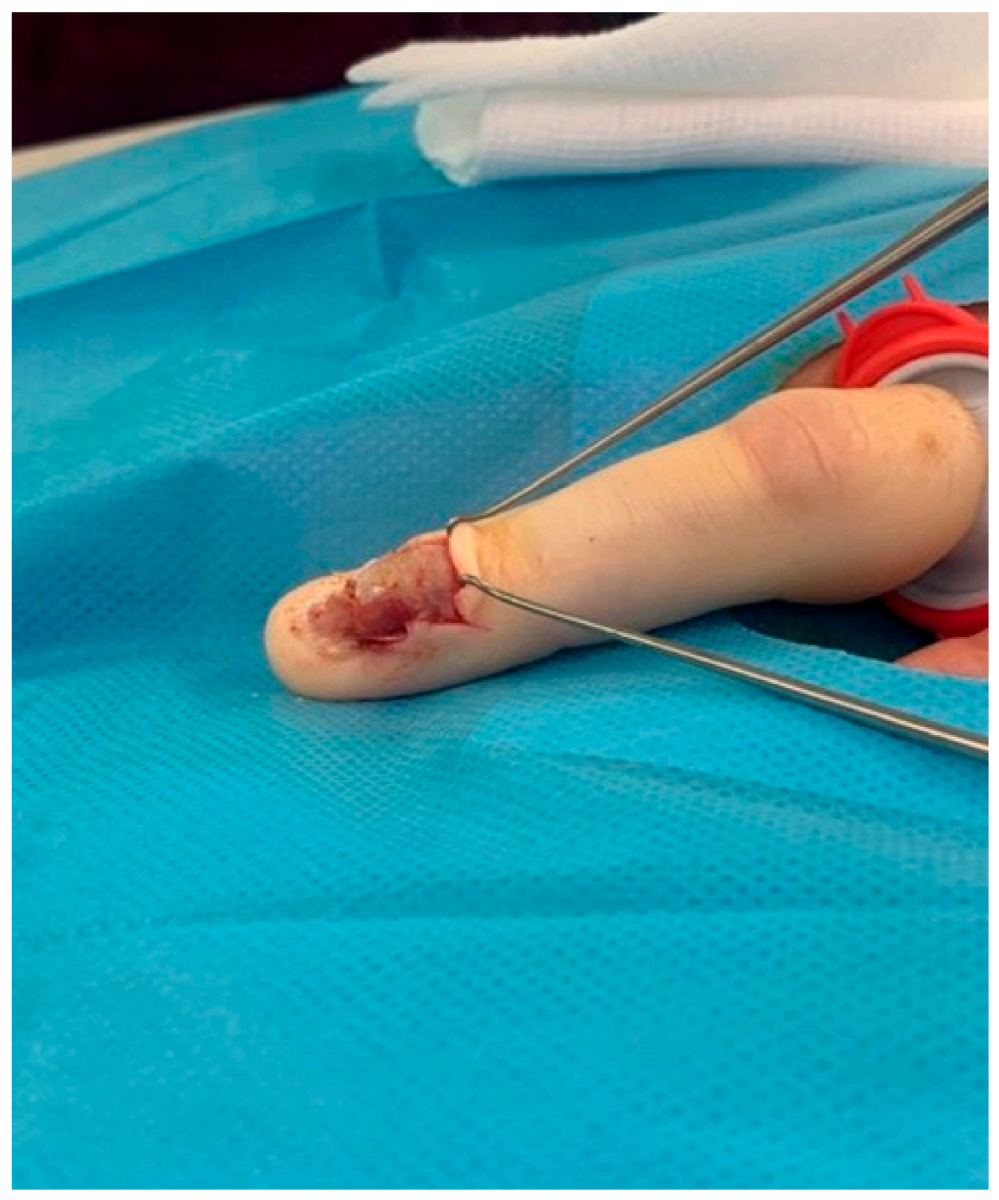
Clinical photograph demonstrating a case of acral melanoma affecting the nail bed of an index finger. This tumour involves both the germinal and sterile matrix of the nail apparatus. In this photograph the nail plate has been removed, Zadek-style incisions have been made at the proximal edge of the nail bed, and the eponychial fold has been reflected to fully expose the area of concern.

**Figure 3:**
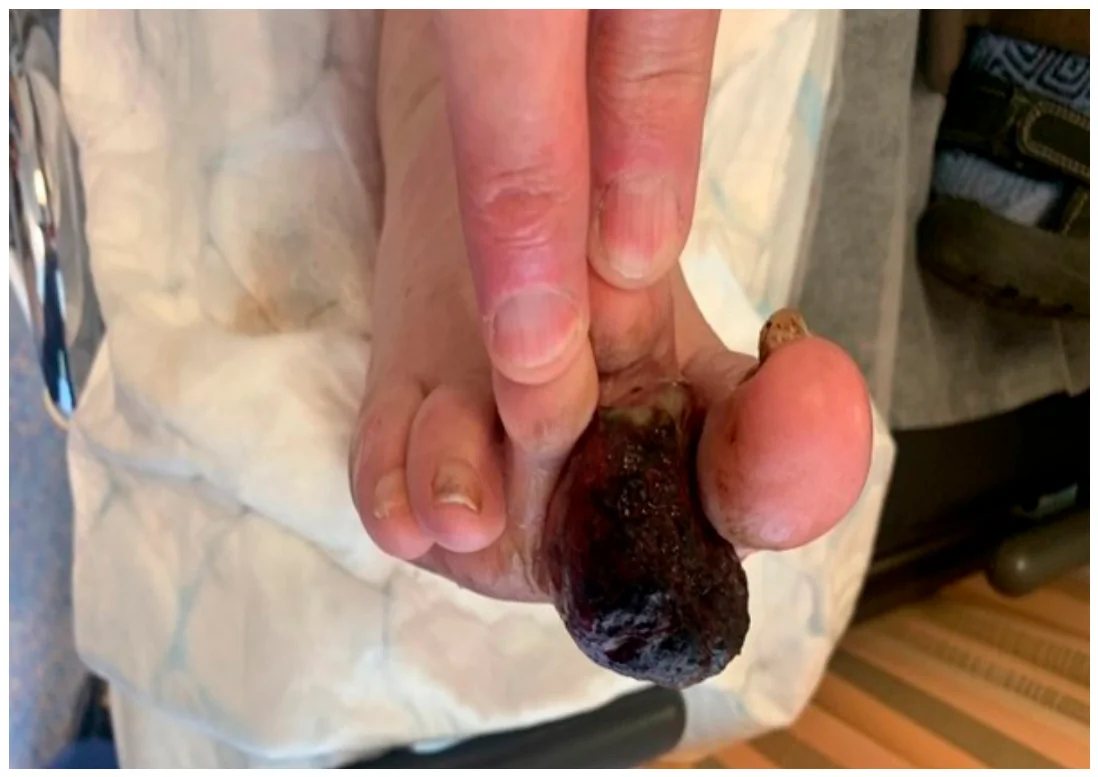
Clinical photograph demonstrating an ulcerated, exophytic lesion located on the plantar aspect of the forefoot. Following histological analysis, this lesion proved to be an acral melanoma.

**Figure 4:**
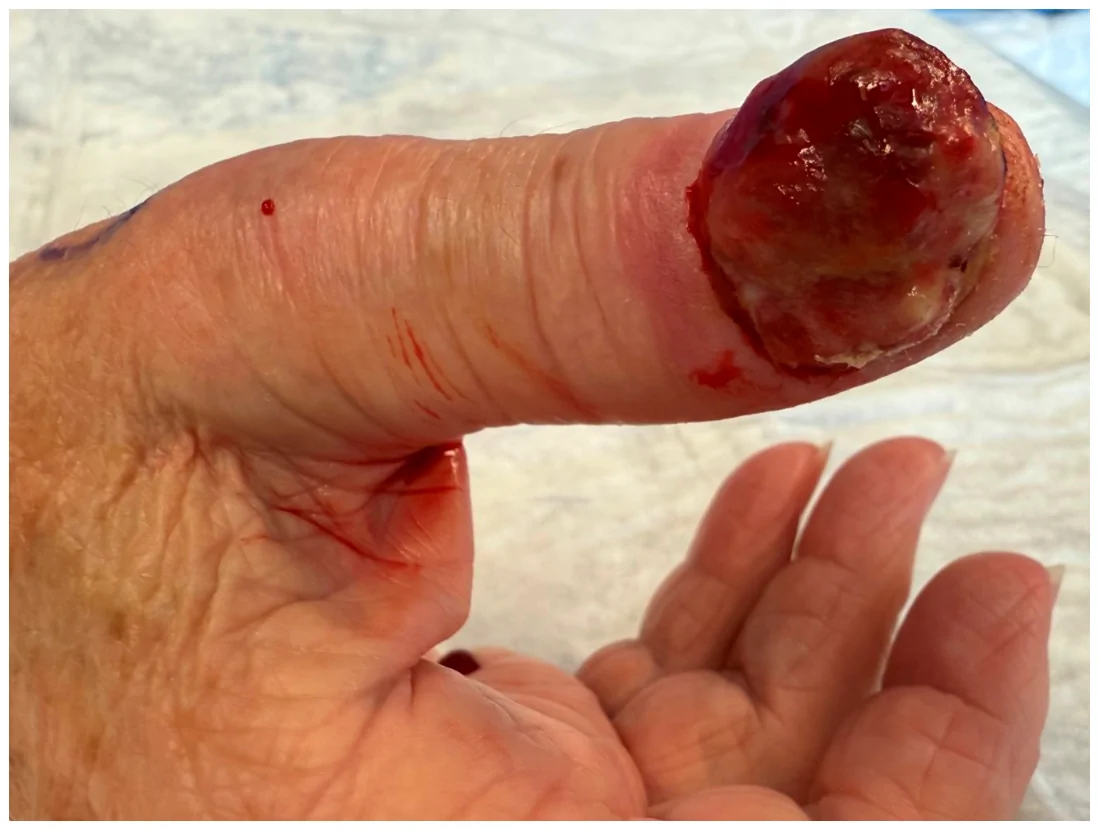
Clinical photograph demonstrating an ulcerated, exophytic lesion involving the germinal and sterile matrix of a thumb. Following histological analysis, this lesion proved to be an acral melanoma.

**Figure 5:**
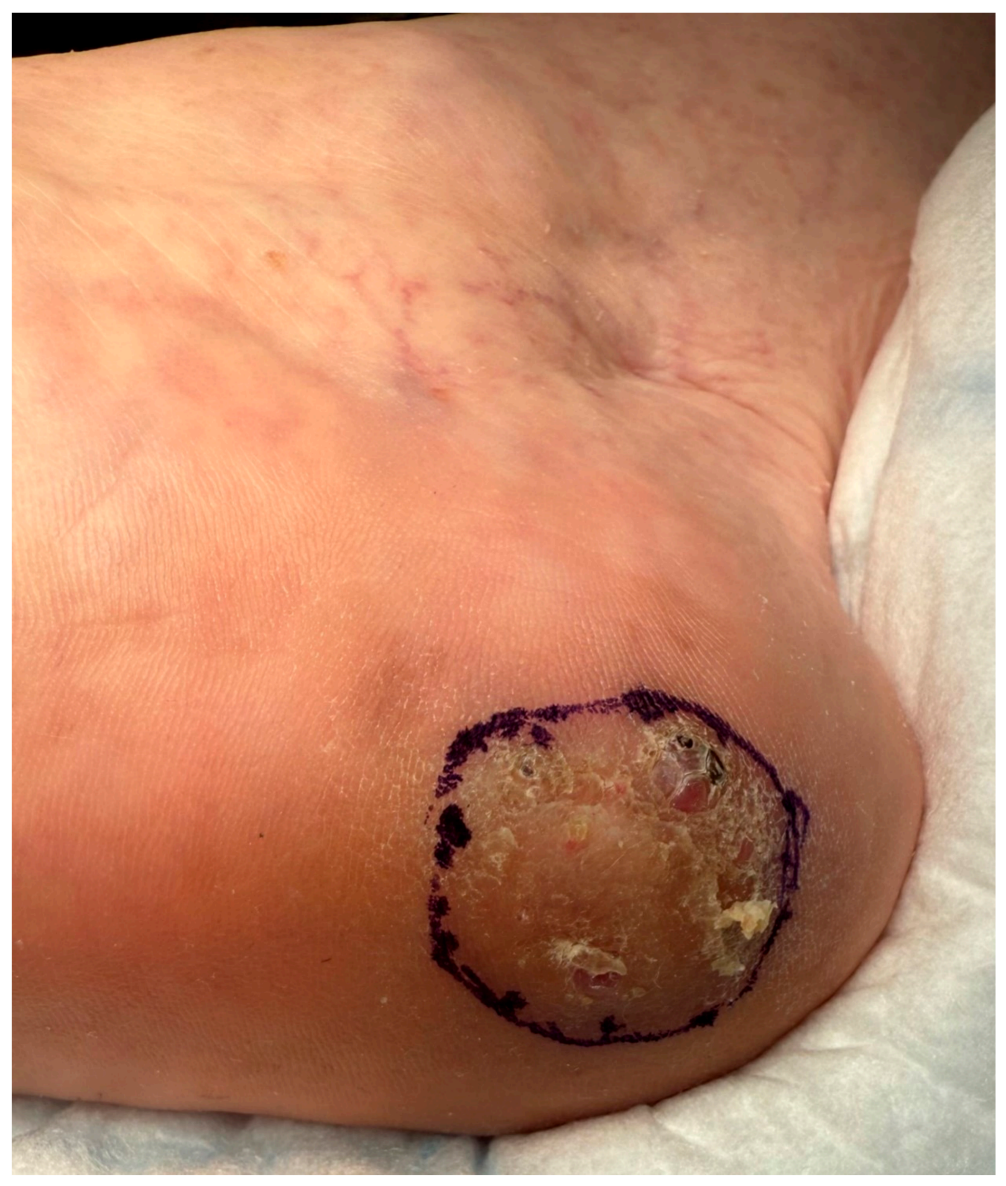
Clinical photograph demonstrating a lesion on the plantar aspect of the hindfoot. Following histological analysis, this lesion proved to be an acral melanoma. The purple boundary represents the clinical delineation of the lesion. The lesion was excised with a wide margin beyond the marked boundary and histological clearance was confirmed.

This study provides the first focused description of AM in an Irish population. Consistent with international Caucasian cohorts [10,17] patients presented at an advanced stage, with a median BT of 3 mm, and 75.6% of tumours staged T3 or higher at diagnosis. The cohort also reflected the delayed-presentation pattern reported elsewhere, as demonstrated in Table 2, with AM occurring predominantly in older patients (median age 73), most commonly on the plantar foot, and frequently after a prolonged period of lesion awareness before seeking care. This pattern is likely multifactorial, explained by the combination of an inconspicuous anatomical site (Figure 2, Figure 3, Figure 4 and Figure 5), an older patient population in whom lesions may be attributed to diabetic ulceration, tinea pedis, a pressure area, or minor trauma [5], and a substantial proportion of clinically amelanotic tumours [18,19], which is itself associated with a worse prognosis [20]. Forty-one percent of patients in our cohort presented with a lesion lacking macroscopic pigment, a higher rate than reported in the literature, with Phan et al. observing absent macroscopic pigment in 27% of acral melanoma cases [10].

### 4.2. Diagnostic Recognition of Acral Melanoma

The ABCDE algorithm, while a useful triage tool for pigmented lesions generally, performs poorly for AM because of the atypical locations, rarity, and frequent lack of pigmentation [5], as seen in our cohort (Figure 2, Figure 3, Figure 4 and Figure 5). Two structured alternatives have been developed specifically for this purpose [5]. The CUBED acronym (Coloured, Uncertain diagnosis, Bleeding, Enlarging, Deteriorating or delayed healing) flags lesions with two or more features as warranting specialist referral [5]. The dermoscopic BRAAFF checklist [21] incorporates irregular blotches, asymmetry of structures and colours, and the presence of a parallel ridge pattern (PRP), described as pigmentation accentuated along the skin ridges rather than the furrows. This PRP is considered the dermoscopic hallmark of AM, distinguishing it from benign acral naevi, which typically show a furrow pattern [21]. In this cohort, prolonged lesion awareness before presentation suggests that diagnostic delay reflects both patient-level delay and challenges in clinical recognition. While improved familiarity with these tools among first-contact clinicians may help, greater public awareness of persistent acral lesions is also required.

### 4.3. Biopsy and Surgical Management

**Figure 6:**
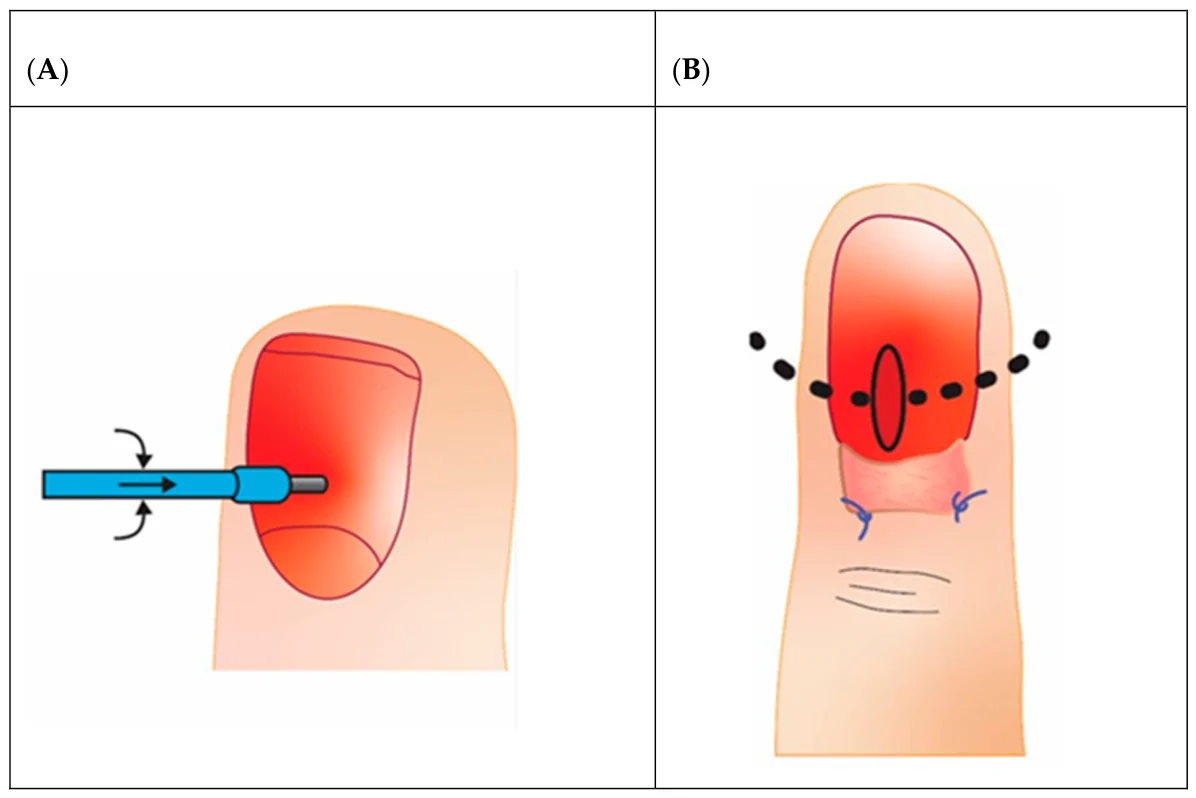
Illustration demonstrating the appropriate longitudinal incisional biopsy for suspected cases of melanoma affecting the nail unit. The image on the left illustrates the traditional approach involving punch biopsy of the nail bed and plate, which is no longer recommended [18]. The image on the right demonstrates correct method of removing the nail plate, making a Zadek-style incision, and reflecting the eponychial fold (dashed line), to perform a combined incisional biopsy of the germinal and sterile matrix, and the underlying periosteum.

Once a suspicious lesion has been identified, prompt referral for dermoscopic assessment and biopsy is recommended [22]. For suspected cutaneous melanoma, an excisional biopsy incorporating a 2 mm margin of normal skin and a cuff of subcutaneous fat is recommended [23]. However, biopsy of a pigmented nail lesion requires a different approach. Melanocytes are now recognised to be present in the nail bed (sterile) matrix as well as the germinal matrix [24]. Therefore, the recommended technique is a longitudinal incisional biopsy following nail plate removal and a Zadek incision to reflect the proximal nail fold, with the nail plate also submitted for histological assessment (Figure 6) [25].

Local disease control in AM frequently requires digital or more proximal amputation, leading to significant functional and psychosocial consequences [26]. This pattern was reflected in this cohort, where 65.9% of patients required amputation. For selected subungual cases, digit-sparing surgery (DSS) has been suggested as a function-preserving alternative. While prospective data are limited, retrospective data suggest comparable melanoma-specific survival, although local recurrence may be more frequent in DSS [26,27]. Given the high rate of amputation seen in this cohort, case-by-case consideration of DSS in appropriately selected patients [18] may help reduce this functional burden [8] while further long-term oncological evidence accrues.

### 4.4. Molecular Features and Emerging Systemic Therapies

Nine of the tumours (39.1%) with mutation data available in this cohort were TWT, consistent with the higher rate of TWT melanoma reported in AM relative to other cutaneous melanoma subtypes [6]. This is likely due to AM’s weaker association with UV-induced damage and lower tumour mutational burden compared with non-acral melanoma [6]. Whole genome sequencing of TWT acral melanoma may therefore be useful to identify alternative oncogenes, including alterations in CDK4, TERT, and cyclin D1, and to inform future targeted therapeutic strategies [28]. These molecular differences may also have implications for systemic therapy, as the absence of common driver mutations may limit standard targeted treatment options. Although immunotherapy has transformed melanoma treatment, response rates in AM appear lower than in non-acral melanoma patients [29], potentially due to this lower tumour mutational burden and distinct tumour biology. Nevertheless, contemporary trials such as NADINA demonstrate the increasing role of neoadjuvant immunotherapy in resectable stage III melanoma, including in BRAF-wild-type disease [30]. While these data are not AM-specific, they support continued evaluation of immunotherapy and molecularly informed treatment strategies for AM.

The Irish population has a comparatively high prevalence of MC1R variants and previously reported lower rate of BRAF mutations in cutaneous melanoma compared with international cohorts [11,12,13]. MC1R status was not assessed in the present cohort, and no association between MC1R and BRAF status could therefore be examined. However, the mutational profile observed in this AM cohort, characterised by low frequencies of BRAF, NRAS, and KIT mutations, and a high proportion of TWT tumours, is broadly in keeping with international AM data [14,31,32], despite this distinctive germline background. We therefore hypothesise that AM’s UV-independent biology, rather than population-level germline background, is the principal determinant of its mutational landscape. The distinction between AM-specific and population-level mutational patterns has not been previously highlighted in an Irish cohort and underscores the importance of describing melanoma subtypes within distinct populations rather than extrapolating from heterogeneous international data.

### 4.5. Strengths and Limitations

This study’s principal strength is that it provides the first dedicated, bi-institutional description of AM in an Irish population, with a catchment covering close to 40% of the national population. The use of two tertiary melanoma referral centres allowed the capture of a clinically meaningful cohort of this rare melanoma subtype and enabled detailed description of demographic, clinicopathological, molecular, treatment-related, and outcome features within a defined Irish setting. However, its principal limitations are those inherent to a small cohort of a rare tumour. The small sample size (*n* = 41), and smaller subset with mutation testing available, limited statistical power and precluded robust multivariable analysis. Furthermore, dermoscopy findings were not recorded, and clarification of the intra-acral subsite (nail apparatus or glabrous skin) was unavailable in seven patients, and outcome data were unavailable for six patients, precluding melanoma-specific survival analysis. In addition, MC1R status was not assessed, so the relationship between germline background and the observed mutational profile could not be directly examined. A structured national melanoma registry capturing dermatopathological, clinical, molecular and outcome data, such as ADOREG in Germany [33], or dedicated biobanks such as BioMEL in Sweden [34], may support more robust future studies. Nevertheless, the study provides a useful baseline description of AM in Ireland and identifies clinically relevant patterns in diagnosis, management and tumour biology.

## 5. Conclusions

Acral melanoma in this Irish cohort presented at an advanced stage, with high rates of amputation, nodal involvement, and disease recurrence, broadly mirroring the poor prognosis reported for AM internationally. Delayed presentation, an inconspicuous anatomical distribution, and a high proportion of clinically amelanotic tumours are the most plausible contributors to this pattern. Wider use of acral-specific diagnostic tools (CUBED, BRAAFF), standardised longitudinal incisional biopsy technique for nail-unit lesions, case-by-case consideration of digit-sparing surgery, and the development of a national melanoma registry with structured molecular data capture represent practical steps that could support earlier recognition and more consistent management of this rare, high-risk melanoma subtype in Ireland.

## Figures and Tables

**Table 1:** Clinicopathological characteristics of patients included in the study.

Sex	
Male, No. (%)	19 (46.34)
Female, No. (%)	22 (53.66)
Age at Diagnosis	
Median (Range)	73 (39–88)
Mean (SD)	70.15 (12.37)
Tumour Location, No. (%)	
Plantar Foot	14 (34.15)
Hallux	9 (21.95)
Toe (excluding hallux)	6 (14.63)
Forefoot	1 (2.44)
Foot Total	30 (73.17)
Thumb	4 (9.76)
Finger (excluding thumb)	6 (14.63)
Hypothenar Eminence	1 (2.44)
Hand Total	11 (26.83)
Acral Subsite, No. (%)	
Plantar/volar (glabrous skin)	17 (41.46)
Nail Apparatus/subungual	11 (26.83)
Acral Digit, Site Not Specified	13 (31.71)
Histological Subtype, No. (%)	
Acral Lentiginous Melanoma	34 (82.93)
Nodular Melanoma	5 (12.20)
Superficial Spreading Melanoma	1 (2.44)
Spindle Cell Melanoma	1 (2.44)
Pigmentation, No. (%)	
Yes	24 (58.54)
No	17 (41.46)
T Stage, No. (%)	
1a	2 (4.89)
1b	2 (4.89)
2a	5 (12.20)
2b	1 (2.44)
3a	5 (12.20)
3b	7 (17.07)
4a	7 (17.07)
4b	12 (29.29)
Breslow Thickness, mm, No. (%)	
≤1	5 (12.20)
>1–2	6 (14.63)
>2–4	12 (29.29)
>4	18 (43.90)
Ulceration. No. (%)	
Yes	17 (41.46)
No	22 (53.66)
PNI, No. (%)	
Yes	6 (14.63)
No	34 (82.93)
LVI, No. (%)	
Yes	8 (19.51)
No	32 (78.05)
Mitotic Rate, per mm^2,^ Median (range)	3.0 (0–40)
Microsatellites, No. (%)	5 (12.2)
Regression, No. (%)	2
Mutation Status (% of 23 cases that underwent mutation analysis), No. (%)	
BRAF	7 (30.43)
NRAS	5 (21.74)
KIT	4 (17.39)
None (Triple-Wild-Type)	9 (39.14)
Not Tested	18
Nodal Biopsy Status, No. (%)	
Positive SLNBx	15 (36.59)
Positive Core Biopsy of Palpable Nodes	3 (7.32)
Negative SLNBx	20 (48.78)
No Nodal Biopsy	3 (7.32)
Recurrence, No. (%)	
Yes	15 (36.59)
No	26 (63.41)
Received Immunotherapy, No. (%)	
Yes	12 (29.27)
No	29 (70.73)

**Table 2:** Comparison with published acral melanoma cohorts.

Feature	This Study (Ireland, *n* = 41)	Phan et al. [10] (France, *n* = 126)	Angel-Baldo et al. [17]
Setting	Two tertiary centres	Single referral centre	20-hospital multicentre
Age, years	73 (median)	63 (median)	67.5 (median)
Nail/subungual %	26.83%	37%	NR
Breslow Thickness (mm)	3.0 (median)	1.45 (median)	2.7 (median)
Acral Lentiginous Melanoma Subtype, %	82.93%	100%	88.4%
Non-pigmented	41.46%	28%	NR
BRAF mutation, %	30.43%	NR	NR
Triple-Wild-Type, %	39.14%	NR	NR
Ulcerated, %	41.46%	NR	47.7%
Positive SLNB, %	36.59% ^1^	NR	27.6% ^1^
Recurrence	36.6% (8–85-month follow-up)	14% (2.9-year median follow-up)	61.8% (5-year recurrence-free survival)

^1^ Positive SLNB is expressed as a percentage of the 35 patients who underwent sentinel lymph node biopsy in both cohorts.

## Data Availability

The data presented in this study are available on request from the corresponding author due to privacy and ethical restrictions on the sharing of identifiable clinical data.

## Supplementary Materials

The following supporting information can be downloaded at: https://www.mdpi.com/article/10.3390/dermatopathology13040046/s1, STROBE Statement—checklist of items that should be included in reports of observational studies.

## Abbreviations

The following abbreviations are used in this manuscript:

AJCC	American Joint Committee on Cancer
ALM	Acral Lentiginous Melanoma
AM	Acral Melanoma
BT	Breslow Thickness
DSS	Digit-Sparing Surgery
HR	Hazard Ratio
LVI	Lymphovascular Invasion
MC1R	Melanocortin-1 Receptor
NM	Nodular Melanoma
OS	Overall Survival
PNI	Perineural Invasion
PRP	Parallel Ridge Pattern
SD	Standard Deviation
SLNBx	Sentinel Lymph Node Biopsy
SSM	Superficial Spreading Melanoma
TWT	Triple-Wild-Type
UV	Ultraviolet
