# Supplementary material for: Acral Melanoma in an Irish Population: Clinicopathological Features, Molecular Profile and Implications for Early Detection
Source: Dermatopathology (Basel). 2026 Oct 1;13(4):46. doi: 10.3390/dermatopathology13040046 (PMC13692538; doi:10.3390/dermatopathology13040046)
Supplement: Supplementary file 1 [file dermatopathology-13-00046-s001.zip › dermatopathology-4512323-supplementary.pdf]

Acral Melanoma in an Irish Population: Clinicopathological Features, Molecular Profile and Implications for Early Detection

Mary Ellen McMahon [1], Stephanie M. Bollard [2,*], John M. Keaney [3], Linda Kelly [3], Silvia Marino [2], Michael O'Connor [3], Chloe Spillane [3], Donal Murphy [1], Aurelie Fabre [4,5], Barry O'Sullivan [1,3], Arnold D.K. Hill [3,6], Fiachra Martin [1] and Roisin T. Dolan [2,5]

STROBE Statement—checklist of items that should be included in reports of observational studies

	Item No.	Recommendation	Page No.	Relevant text from manuscript
**Title and abstract**	1	(*a*) Indicate the study's design with a commonly used term in the title or the abstract	1	"retrospective, bi-institutional cohort study"
		(*b*) Provide in the abstract an informative and balanced summary of what was done and what was found	1	Structured abstract
**Introduction**				
Background/rationale	2	Explain the scientific background and rationale for the investigation being reported	2-3	Introduction, paragraphs 1-4
Objectives	3	State specific objectives, including any prespecified hypotheses	3	Introduction, paragraph 4 "The aims were to characterise…."
**Methods**				
Study design	4	Present key elements of study design early in the paper	3	Methods 2.1
Setting	5	Describe the setting, locations, and relevant dates, including periods of recruitment, exposure, follow-up, and data collection	3, 6	Methods 2.1 (two Dublin tertiary centres from 2017 - 2023, from prospective melanoma databases, medical records and radiology/histopathology reports. Median follow-up reported Section 3.3.
Participants	6	(*a*) *Cohort study*—Give the eligibility criteria, and the sources and methods of selection of participants. Describe methods of follow-up		Methods 2.2 – Inclusion histologically confirmed AM,

		Case-control study—Give the eligibility criteria, and the sources and methods of case ascertainment and control selection. Give the rationale for the choice of cases and controls<br>*Cross-sectional study*—Give the eligibility criteria, and the sources and methods of selection of participants		no additional exclusion criteria applied. Follow-up per stage standard appropriate protocol (clinical review with imaging where indicated)
		(*b*) *Cohort study*—For matched studies, give matching criteria and number of exposed and unexposed<br>*Case-control study*—For matched studies, give matching criteria and the number of controls per case		Not applicable
Variables	7	Clearly define all outcomes, exposures, predictors, potential confounders, and effect modifiers. Give diagnostic criteria, if applicable	3	Methods 2.2
Data sources/ measurement	8*	For each variable of interest, give sources of data and details of methods of assessment (measurement). Describe comparability of assessment methods if there is more than one group	3	Methods 2.2 – prospectively maintained databases, medical records, radiology/histopathology reports.
Bias	9	Describe any efforts to address potential sources of bias	3	Methods 2.1 – "As cutaneous melanoma is managed only in specialist tertiary referral centres in our jurisdiction, case capture is expected to be comprehensive, with selection bias considered to be limited."
Study size	10	Explain how the study size was arrived at	3	Methods 2.1 – "No formal sample size calculation was performed. All patients meeting the inclusion criteria in the study period were included."

Continued on next page

Quantitative variables	11	Explain how quantitative variables were handled in the analyses. If applicable, describe which groupings were chosen and why	3, 4	Methods 2.3/Table 1 - Breslow thickness and tumour stage categorised per AJCC 8th-edition thresholds.
Statistical methods	12	(*a*) Describe all statistical methods, including those used to control for confounding	3	Methods 2.3. (a) Chi-squared / non-parametric tests; Cramer's V for strength of association; SPSS v29.
		(*b*) Describe any methods used to examine subgroups and interactions	3	(b) Mutation-status subgroup described – descriptive only
		(*c*) Explain how missing data were addressed	3	(c) Percentages calculated on available-data denominator. missing values not imputed. – Methods 2.3
		(*d*) *Cohort study*—If applicable, explain how loss to follow-up was addressed<br>*Case-control study*—If applicable, explain how matching of cases and controls was addressed<br>*Cross-sectional study*—If applicable, describe analytical methods taking account of sampling strategy	3	(d) Follow-up as per clinical protocol – Methods 2.2
		(*e*) Describe any sensitivity analyses	3	(e) None performed.
**Results**				
Participants	13*	(a) Report numbers of individuals at each stage of study—eg numbers potentially eligible, examined for eligibility, confirmed eligible, included in the study, completing follow-up, and analysed	4	Results 3.1-3.2/Table 1
		(b) Give reasons for non-participation at each stage	3,4	Missing data indicated in Table 1/Results 3.2
		(c) Consider use of a flow diagram		Not applicable given single stage eligibility
Descriptive data	14*	(a) Give characteristics of study participants (eg demographic, clinical, social) and information on exposures and potential confounders	4	Results 3.1-3.2
		(b) Indicate number of participants with missing data for each variable of interest	4	Missing data indicated in Table 1/Results 3.2

		(c) *Cohort study*—Summarise follow-up time (eg, average and total amount)	5	Results 3.3 – Treatment & outcomes (median follow-up 45 months)
Outcome data	15*	*Cohort study*—Report numbers of outcome events or summary measures over time	*4,5*	Results 3.3/Table 1
		Case-control study—Report numbers in each exposure category, or summary measures of exposure		*Not applicable*
		Cross-sectional study—Report numbers of outcome events or summary measures		*Not applicable*
Main results	16	(*a*) Give unadjusted estimates and, if applicable, confounder-adjusted estimates and their precision (eg, 95% confidence interval). Make clear which confounders were adjusted for and why they were included	4,5	Results 3.2, 3.3 – Descriptive study ,no adjustments – as per sample size limitation.
		(*b*) Report category boundaries when continuous variables were categorized	4,5	Table 1 – AJCC 8th edition boundaries
		(*c*) If relevant, consider translating estimates of relative risk into absolute risk for a meaningful time period		Not applicable

Continued on next page

Other analyses	17	Report other analyses done—eg analyses of subgroups and interactions, and sensitivity analyses		Results 3.2 – descriptive reporting of mutation status subgroup
**Discussion**				
Key results	18	Summarise key results with reference to study objectives	6	Discussion 4.1
Limitations	19	Discuss limitations of the study, taking into account sources of potential bias or imprecision. Discuss both direction and magnitude of any potential bias	11	Discussion 4.6
Interpretation	20	Give a cautious overall interpretation of results considering objectives, limitations, multiplicity of analyses, results from similar studies, and other relevant evidence	6-11	Discussion 4.1 – 4.6
Generalisability	21	Discuss the generalisability (external validity) of the study results	11	Discussion 4.6 – relevance of 30% of national population
**Other information**				
Funding	22	Give the source of funding and the role of the funders for the present study and, if applicable, for the original study on which the present article is based	11	Funding statement – This research received no external funding

*Give information separately for cases and controls in case-control studies and, if applicable, for exposed and unexposed groups in cohort and cross-sectional studies.

Note: An Explanation and Elaboration article discusses each checklist item and gives methodological background and published examples of transparent reporting. The STROBE checklist is best used in conjunction with this article (freely available on the Web sites of PLoS Medicine at http://www.plosmedicine.org/, Annals of Internal Medicine at http://www.annals.org/, and Epidemiology at http://www.epidem.com/). Information on the STROBE Initiative is available at www.strobe-statement.org.
